# Prognostically relevant periprocedural myocardial injury and infarction associated with percutaneous coronary interventions: a Consensus Document of the ESC Working Group on Cellular Biology of the Heart and European Association of Percutaneous Cardiovascular Interventions (EAPCI)

**DOI:** 10.1093/eurheartj/ehab271

**Published:** 2021-05-31

**Authors:** Heerajnarain Bulluck, Valeria Paradies, Emanuele Barbato, Andreas Baumbach, Hans Erik Bøtker, Davide Capodanno, Raffaele De Caterina, Claudio Cavallini, Sean M Davidson, Dmitriy N Feldman, Péter Ferdinandy, Sebastiano Gili, Mariann Gyöngyösi, Vijay Kunadian, Sze-Yuan Ooi, Rosalinda Madonna, Michael Marber, Roxana Mehran, Gjin Ndrepepa, Cinzia Perrino, Stefanie Schüpke, Johanne Silvain, Joost P G Sluijter, Giuseppe Tarantini, Gabor G Toth, Linda W Van Laake, Clemens von Birgelen, Michel Zeitouni, Allan S Jaffe, Kristian Thygesen, Derek J Hausenloy

**Affiliations:** Department of Cardiology, Norfolk and Norwich University Hospital, Colney Lane, Norwich, Norfolk, NR4 7UY, UK; Norwich Medical School, Bob Champion Research and Educational Building, Rosalind Franklin Road, University of East Anglia, Norwich Research Park. Norwich, Norfolk, NR4 7UQ, United Kingdom; Cardiology Department, Maasstad Hospital, Maasstadweg 21, 3079 DZ Rotterdam, The Netherlands; Department of Advanced Biomedical Sciences, Federico II University, Via Pansini 5, 8013, Naples, Italy; Cardiovascular Center Aalst OLV Hospital, Moorselbaan n. 164, 9300 Aalst, Belgium; Centre for Cardiovascular Medicine and Devices, William Harvey Research Institute, Queen Mary University of London, Barts Heart Centre, Charterhouse Square, London, EC1M 6BQ, UK; Yale University School of Medicine, 333 Cedar St, New Haven, CT 06510, USA; Department of Cardiology, Aarhus University Hospital, Palle Juul-Jensens Boulevard 99, DK-8200 Aarhus N, Denmark; Division of Cardiology, Azienda Ospedaliero-Universitaria Policlinico “G. Rodolico-San Marco”, University of Catania, Via Santa Sofia 78, 95100 Catania, Italy; Department of Pathology, Cardiology Division, University of Pisa, Lungarno Antonio Pacinotti, 43, 56124 Pisa, Italy; University of Pisa, and Cardiology Division, Pisa University Hospital AND Fondazione VillaSerena per la Ricerca, Città Sant’Angelo, Pescara, Italy; Department of Cardiology, Santa Maria della Misericordia Hospital, Piazzale Giorgio Menghini, 1, 06129 Perugia, Italy; The Hatter Cardiovascular Institute, University College London, 67 Chenies Mews London, WC1E 6HX, UK; Division of Cardiology, Weill Cornell Medical College, New York Presbyterian Hospital, 1414 York Ave, New York, NY 10021, USA; Department of Pharmacology and Pharmacotherapy, Semmelweis University, Nagyvarad tér 4, Budapest, 1089 Hungary; Pharmahungary Group, Hajnóczy u. 6, Szeged, 6722 Hungary; Centro Cardiologico Monzino, Istituto di Ricovero e Cura a Carattere Scientifico, Via Carlo Parea, 4, 20138 Milano MI, Italy; Department of Cardiology, Medical University of Vienna, Waehringer Guertel 18-20, Vienna A-1090, Austria; Translational and Clinical Research Institute, Faculty of Medical Sciences, Newcastle University, M4:146 4th Floor William Leech Building, Newcastle University Medical School, Newcastle upon Tyne, NE2 4HH, UK; Freeman Hospital, Newcastle upon Tyne Hospitals NHS Foundation Trust, Cardiothoracic centre, High Heaton, Newcastle upon Tyne, NE7 7DN, UK; Eastern Heart Clinic, Prince of Wales Hospital, Barker St, Randwick NSW 2031, Australia; Department of Pathology, Cardiology Division, University of Pisa, Lungarno Antonio Pacinotti, 43, 56124 Pisa, Italy; Department of Internal Medicine, University of Texas Medical School, Houston, 77060 Houston, TX, USA; School of Cardiovascular Medicine and Sciences, British Heart Foundation Centre of Excellence and National Institute for Health Research Biomedical Research Centre, St. Thomas' Hospital Campus, King's College London, Westminster Bridge Rd, London SE1 7EH, UK; The Zena and Michael A. Wiener Cardiovascular Institute, Icahn School of Medicine at Mount Sinai, 1 Gustave L. Levy Pl, New York, NY 10029, USA; Clinical Trials Center, Cardiovascular Research Foundation, 1700 Broadway, New York, NY 10019, USA; Deutsches Herzzentrum München, Technische Universität, Lazarettstraße 36, 80636 München, Germany; Department of Advanced Biomedical Sciences, Federico II University, Via Pansini 5, 8013, Naples, Italy; Deutsches Herzzentrum München, Lazarettstr. 36, 80636 Munich, Germany; Sorbonne Université, ACTION Study Group, Institut de Cardiologie, Hôpital Pitié-Salpêtrière (AP-HP), INSERM UMRS, Paris 1166, France; Laboratory of Experimental Cardiology, Department of Cardiology, University Medical Center Utrecht, Heidelberglaan 100, 3584 CX, Utrecht, The Netherlands; Regenerative Medicine Center Utrecht, Circulatory Health Laboratory, University Utrecht, University Medical Center Utrecht, Heidelberglaan 100, 3584 CX, Utrecht, The Netherlands; Interventional Cardiology, Department of Cardiac, Thoracic, Vascular Sciences and Public Health, University of Padua, Via Giustiniani, 2 – 35128 Padova, Italy; University Heart Center Graz, Division of Cardiology, Department of Medicine, Medical University Graz, Auenbruggerplatz 15, 8036 Graz, Austria; Division Heart and Lungs, Department of Cardiology and Regenerative Medicine Center, University Medical Center Utrecht, Heidelberglaan 100, 3574 CX Utrecht, The Netherlands; Department of Cardiology, Thoraxcentrum Twente, Medisch Spectum Twente, Koningstraat 1, 7512 KZ Enschede, The Netherlands; Department of Health Technology and Services Research, Faculty BMS, Technical Medical Centre, University of Twente, Hallenweg 5, 7522 NH Enschede, The Netherlands; Sorbonne Université, ACTION Study Group, Institut de Cardiologie, Hôpital Pitié-Salpêtrière (AP-HP), INSERM UMRS, Paris 1166, France; Departments of Cardiology and Laboratory Medicine and Pathology, Mayo Clinic, 200 First St SW, Rochester, MN, 55905, USA; Department of Cardiology, Aarhus University Hospital, Palle Juul-Jensens Boulevard 99, DK-8200 Aarhus N, Denmark; The Hatter Cardiovascular Institute, University College London, 67 Chenies Mews London, WC1E 6HX, UK; Cardiovascular and Metabolic Disorders Program, Duke-National University of Singapore, 8 College Road, Singapore 169857, Singapore; National Heart Research Institute Singapore, National Heart Centre, 5 Hospital Drive, Singapore 169609, Singapore; Yong Loo Lin School of Medicine, National University Singapore, 1E Kent Ridge Road, Singapore 119228, Singapore; Cardiovascular Research Center, College of Medical and Health Sciences, Asia University, 500, Lioufeng Rd., Wufeng, Taichung 41354, Taiwan

**Keywords:** Percutaneous coronary intervention, Periprocedural myocardial injury, Periprocedural myocardial infarction, Type 4a myocardial infarction, Chronic coronary syndrome

## Abstract

A substantial number of chronic coronary syndrome (CCS) patients undergoing percutaneous coronary intervention (PCI) experience periprocedural myocardial injury or infarction. Accurate diagnosis of these PCI-related complications is required to guide further management given that their occurrence may be associated with increased risk of major adverse cardiac events (MACE). Due to lack of scientific data, the cut-off thresholds of post-PCI cardiac troponin (cTn) elevation used for defining periprocedural myocardial injury and infarction, have been selected based on expert consensus opinions, and their prognostic relevance remains unclear. In this Consensus Document from the ESC Working Group on Cellular Biology of the Heart and European Association of Percutaneous Cardiovascular Interventions (EAPCI), we recommend, whenever possible, the measurement of baseline (pre-PCI) cTn and post-PCI cTn values in all CCS patients undergoing PCI. We confirm the prognostic relevance of the post-PCI cTn elevation >5× 99th percentile URL threshold used to define type 4a myocardial infarction (MI). In the absence of periprocedural angiographic flow-limiting complications or electrocardiogram (ECG) and imaging evidence of new myocardial ischaemia, we propose the same post-PCI cTn cut-off threshold (>5× 99th percentile URL) be used to define prognostically relevant ‘major’ periprocedural myocardial injury. As both type 4a MI and major periprocedural myocardial injury are strong independent predictors of all-cause mortality at 1 year post-PCI, they may be used as quality metrics and surrogate endpoints for clinical trials. Further research is needed to evaluate treatment strategies for reducing the risk of major periprocedural myocardial injury, type 4a MI, and MACE in CCS patients undergoing PCI.

## Graphical Abstract

**Figure:**
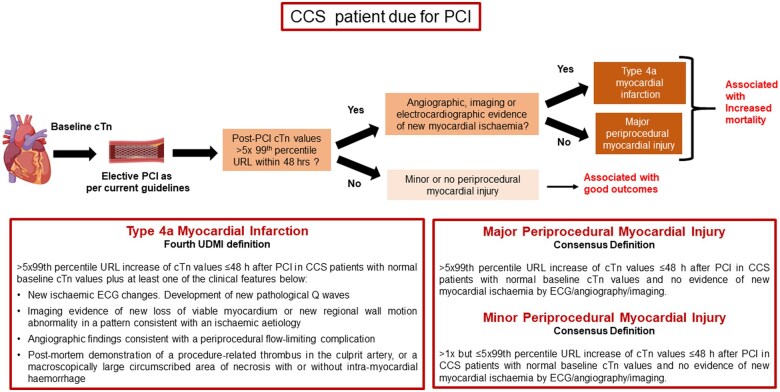
An overview of the suggested approach to diagnosing the presence of ‘minor’ and prognostically relevant ‘major’ periprocedural myocardial injury (as defined in this Consensus document) and type 4a myocardial infarction (as defined by the 4th Universal Definition of Myocardial Infarction) in chronic coronary syndrome patients undergoing percutaneous coronary intervention. CCS, chronic coronary syndrome; MI, myocardial infarction; PCI, percutaneous coronary intervention.

## Introduction

Percutaneous coronary intervention (PCI) remains the major revascularization strategy for patients with obstructive coronary artery disease (CAD), with an estimated 5 million procedures performed worldwide each year.^1^ In a substantial number of PCI cases for acute coronary syndrome (ACS) and chronic coronary syndrome (CCS),^2^ periprocedural myocardial injury or myocardial infarction (MI) occurs,^3^ the actual incidences of which depend on the cardiac biomarker measured and the definitions used. Both these PCI-related complications may be associated with an increased risk of future major adverse cardiovascular events (such as death, re-infarction, and revascularization).^3^^,^^4^ Due to lack of scientific data, the cut-off thresholds of post-PCI elevations of cardiac troponin (cTn) values used for defining periprocedural myocardial injury and MI have been based on expert consensus opinions.^5–7^ As such, evidence-based cut-off thresholds of post-PCI cTn elevations for defining prognostically relevant periprocedural myocardial injury and MI need to be established. This is particularly important given the use of periprocedural MI as part of the primary composite endpoint in recent clinical trials of CCS patients undergoing PCI.^8–11^ Furthermore, the choice of periprocedural MI definition has been shown to influence the outcomes in recent clinical trials including ISCHEMIA,^12^^,^^13^ SYNTAXES,^14^ and EXCEL.^15^

In this Consensus Document by the European Society of Cardiology (ESC) Working Group on Cellular Biology of the Heart and the European Association of Percutaneous Cardiovascular Interventions (EAPCI), we review the latest scientific data evaluating the prognostic relevance of post-PCI cTn elevations. We have restricted our focus to CCS patients undergoing PCI with normal baseline or elevated but stable baseline (pre-PCI) cTn values, although periprocedural myocardial injury and type 4a MI are of course also relevant to ACS patients undergoing urgent PCI. The aims of our Consensus Document are as follows: (i) establish the cut-off thresholds of post-PCI cTn elevations for defining prognostically relevant periprocedural myocardial injury and type 4a MI; (ii) determine the incidences of periprocedural myocardial injury and type 4a MI; (iii) identify the patient features, lesion characteristics, and periprocedural factors, which independently predict future major adverse cardiac events (MACE); and (iv) provide recommendations for the diagnosis of periprocedural myocardial injury or type 4a MI.

## Defining periprocedural myocardial infarction and injury

A number of different diagnostic criteria have been proposed to define periprocedural MI (*Table 1*, Supplementary material online, *Table S1*).^6^^,^^7^^,^^16–20^ Whereas the Universal Definition of Myocardial Infarction (UDMI) task force has based the definition of type 4a MI on relatively low thresholds of cardiac biomarker elevations together with the presence of new myocardial ischaemia, the Society for Cardiovascular Angiography and Interventions (SCAI)^21^ and Academic Research Consortium-2 (ARC-2)^6^ have proposed higher thresholds of cardiac biomarker elevation to define periprocedural MI. More centres are changing from conventional cTn to high-sensitivity cTn (hs-cTn) assays, and the latter have been used to define periprocedural MI.^6^^,^^7^ As expected, the incidence of periprocedural MI in CCS patients varies according to the definition and cardiac biomarker used. For type 4a MI (3rd UDMI), the incidence was 7% with hs-cTnT,^3^ and 10% with cTnT,^22^ whereas for the SCAI definition of periprocedural MI the incidence was only 1.5–2.9%.^3^^,^^23^

**Table 1:** Definitions of periprocedural myocardial injury and infarction in patients with normal baseline (pre-percutaneous coronary intervention) cardiac troponin values

Group	Periprocedural myocardial injury	Periprocedural myocardial infarction
Joint ESC/ACC Myocardial Infarction Redefined Consensus Document First UDMI (2000)^16^	Not available	>1× 99th percentile URL cTn increase
Second UDMI (2007)^17^	>1× 99th percentile URL cTn increase	Type 4a MI >3× 99th percentile URL cTn increase
ARC-1 (2007)^18^	Not available	>3× URL cTn increase
Third UDMI (2012)^19^	>1× 99th percentile URL cTn increase >5× 99th percentile URL cTn increase in the absence of ischaemic, angiographic, or imaging findings.	Type 4a MI >5× 99th percentile URL cTn increase within 48 h of procedure plus at least one of: Evidence of prolonged ischaemia (≥20 min) as demonstrated by prolonged chest pain Ischaemic ST changes or new pathological Q waves Angiography evidence of a flow-limiting complication Imaging evidence of new loss of viable myocardium or new regional wall motion abnormality
SCAI (2014)^20^	Not available	≥70× ULN cTn increase in patients with normal baseline cTn ≥35× ULN cTn increase plus new pathologic Q-waves in ≥2 contiguous leads (or new persistent LBBB).
ARC-2 (2018)^6^	≥70× URL cTn increase within 48 h of procedure	≥35× URL cTn increase within 48 h of procedure with one of below: New significant Q waves or equivalent Flow-limiting angiographic complications New ‘substantial’ loss of myocardium on imaging
Fourth UDMI (2018)^7^	>1× 99th percentile URL increase cTn	Type 4a MI >5× 99th percentile URL cTn increase within 48 h of procedure plus at least one of: New ischaemic ECG changes. Development of new pathological Q waves Imaging evidence of new loss of viable myocardium or new regional wall motion abnormality in a pattern consistent with an ischaemic aetiology Angiographic findings consistent with a periprocedural flow-limiting complication Post-mortem demonstration of a procedure-related thrombus in the culprit artery, or a macroscopically large circumscribed area of necrosis with or without intra-myocardial haemorrhage.

ARC-2, Academic Research Consortium-2; cTn, cardiac troponin; LBBB, left bundle branch block; MI, myocardial infarction; SCAI, Society for Cardiovascular Angiography and Interventions; UDMI, Universal Definition of Myocardial Infarction.

In the absence of ECG, angiography or imaging evidence of new myocardial ischaemia required for the 4th UDMI definition of type 4a MI, periprocedural myocardial injury following PCI, as detected by post-PCI elevation of cTn values, should prompt a search for the underlying aetiology (Aetiology of periprocedural myocardial injury and type 4a myocardial infarction section). As with type 4a MI, there exist a number of different definitions for periprocedural myocardial injury in CCS patients undergoing PCI (*Table 1*, Supplementary material online, *Table S1*). The 4th UDMI^7^ has defined periprocedural myocardial injury as any post-PCI elevation of cTn >1× 99th percentile URL in patients with normal baseline (pre-PCI) values. In contrast, ARC-2 has defined significant periprocedural myocardial injury at a much higher threshold of post-PCI cTn elevation (≥70× 99th percentile URL).^6^ As expected, the incidence of periprocedural myocardial injury varies according to the definition and cardiac biomarker used from as low as 2.9% (according to ARC-2 criteria),^23^ to 20% to 43% with conventional cTnT^24^^,^^25^ and 14% to 52% with conventional cTnI,^26^^,^^27^ to as high as 78% to 85% with hs-cTnT.^28^^,^^29^

- In summary, there is a lack of consensus for defining periprocedural myocardial infarction and injury, with the SCAI and ARC definitions stipulating much higher thresholds of post-PCI cTn elevation when compared with the 4th UDMI.

## Detection of periprocedural myocardial injury and type 4a myocardial infarction associated with percutaneous coronary intervention

### Role of cardiac biomarkers

The most sensitive and specific cardiac biomarkers for detecting periprocedural myocardial injury and type 4a MI are post-PCI elevations of hs-cTnI/T values.^30–32^ The diagnostic performances of hs-cTnI/T are significantly better than conventional cTnI/T,^30^ and abundant cytosolic proteins such as creatine kinase (CK), CK-myocardial band (MB), heart-type fatty acid-binding protein, myoglobin, and glycogen phosphorylase. Hs-cTnT/I also outperform protein biomarkers produced outside the heart such as copeptin, C-reactive protein, sCD40, ST2, and myeloperoxidase.^33^ There are some specific issues to take into consideration with the hs-cTnT/I assays when interpreting baseline (pre-PCI) values. Chronic elevations of hs-cTnT/I values can be present in up to 30% of patients, due to comorbidities and risk factors, such as chronic kidney disease, diabetes, structural heart disease, skeletal muscle disease, malignancies, and advanced age.^34^^,^^35^ Other cardiac-restricted proteins, such as cardiac myosin-binding protein C (cMyC), may challenge hs-cTnT/I,^36^ but these assays are not widely available. Although pre-PCI circulating microRNAs have been shown to predict post-PCI outcomes, such as coronary artery restenosis,^37^^,^^38^ their ability to predict the occurrence of periprocedural myocardial injury and type 4a MI is not known, and they are less sensitive than hs-cTnT/I and cMyC.^39^

- In summary, hs-cTn is the cardiac biomarker of choice for detecting periprocedural myocardial injury and type 4a MI in CCS patients undergoing PCI.

### Role of the ECG

Compared with a pre-procedural ECG, new ischaemic ECG changes such as new ST-elevation at the J-point or new horizontal or downsloping ST-depression in two contiguous leads or new pathological Q waves are one of the requirements to define type 4a MI according to the 4th UDMI.^7^ It should be noted that isolated post-PCI development of new pathological Q waves meets the type 4a MI criteria even if cTn values are elevated and rising but ≤5× 99th percentile URL.^7^ The presence of pre-existing left bundle branch block (LBBB) makes the diagnosis of new ischaemic changes challenging. However, in patients with LBBB, ST-elevation ≥1 mm concordant with the QRS complex in any lead may be an indicator of acute myocardial ischaemia.^7^

- In summary, new ischaemic ST-segment changes and/or pathological Q waves on ECG are one of the key criteria for defining type 4a MI in CCS patients undergoing PCI.

### Role of cardiac imaging

Transthoracic echocardiography is the most accessible and available imaging modality for detecting new loss of viable myocardium or new regional wall motion abnormality (RWMA) as one of the diagnostic imaging criteria for defining type 4a MI. However, its comparative lack of sensitivity makes it challenging to detect type 4a MI. Sensitivity may be improved with use of contrast agents that enhance endocardial visualization,^40^ and with advanced echocardiography imaging modalities, such as tissue Doppler imaging or speckle tracking, which may detect more subtle RWMAs.^41^ Due to limitations in spatial image resolution, it may be challenging to detect type 4a MI using myocardial perfusion scintigraphy (SPECT or PET), unless the area of irreversible myocardial injury is comparatively large. Although contrast-enhanced computed tomography can detect irreversible myocardial injury in ACS patients,^42^^,^^43^ its role in imaging type 4a MI following PCI in CCS patients has not been tested.

Late gadolinium enhancement cardiovascular magnetic resonance (LGE-CMR) is the gold-standard imaging technique for detection and quantification of irreversible myocardial injury. It has been used to detect type 4a MI in CCS and ACS patients and has provided unique insights into the underlying pathophysiology. The median mass of new irreversible myocardial injury detected by LGE-CMR ranges from 0.8^44^ to 5 g,^45^ and new LGE occurs in 16%^46^ to 63%^47^ of CCS patients following PCI, and its presence correlates with post-PCI elevations of CK-MB^48^ and cTn.^49^ It occurs in two distinct patterns^47–51^—new LGE immediately adjacent to the stent, due to minor incidental side-branch occlusion (SBO), and new LGE distal to the stent due to distal coronary embolization of atheromatous material. New LGE on CMR is associated with a 3.1-fold increase in MACE at a median follow-up of 2.9 years,^45^ although only modest correlations have been shown with type 4a MI (according to 2nd UDMI).^44^ Late gadolinium enhancement cardiovascular magnetic resonance imaging may, however, miss the occurrence of periprocedural myocardial injury detected by minor elevations of post-PCI cTn values due to the latter’s higher sensitivity.^52^ Although CMR has higher sensitivity for detection of new loss of viable myocardium as part of the diagnostic criteria of type 4a MI, its use is mainly restricted to research studies because of its limited availability.

- In summary, transthoracic echocardiography is the most accessible and available imaging modality for detecting new loss of viable myocardium or RWMA for defining type 4a MI in CCS patients following PCI, although it lacks sensitivity when compared with other cardiac imaging modalities such as CMR.

### Role of coronary angiography

One of the key criteria for diagnosing type 4a MI in CCS patients following PCI according to the 4th UDMI^7^ is new myocardial ischaemia as evidenced by coronary angiographic findings consistent with periprocedural flow-limiting complications, such as coronary dissection, occlusion of a major epicardial artery, SBO/thrombus, disruption of collateral flow, or distal embolization. Academic Research Consortium-2^6^ has provided detailed criteria for defining flow-limiting coronary angiographic complications in PCI patients with suspected periprocedural MI. Interestingly, PCI complications detected on angiography may not always be associated with cardiac biomarker elevations, and minor elevations in cardiac biomarkers may occur due to plaque disruption and local vessel injury without any obvious coronary angiographic complications. Intravascular imaging modalities may be used to complement coronary angiography findings in understanding the pathophysiology of PCI complications.

- In summary, periprocedural flow-limiting complications on coronary angiography are one of the key criteria for diagnosing type 4a MI in CCS patients undergoing PCI.

## Aetiology of periprocedural myocardial injury and type 4a myocardial infarction

**Figure 1:**
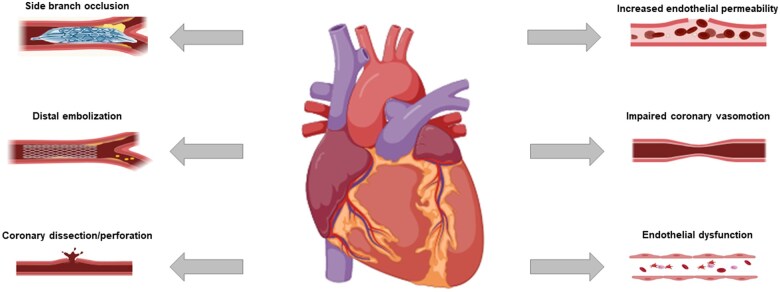
Aetiology of periprocedural myocardial injury and type 4a myocardial infarction.

The aetiology of periprocedural myocardial injury and type 4a MI is multifactorial and may result from PCI-related events or complications, alone or in combination (*Figure 1*). The pathophysiology underlying periprocedural myocardial injury and type 4a MI is inherently very different from type 1 MI. The former is related to the PCI procedure and occurs in the controlled setting of a catheter laboratory, whereas the latter often occurs as an emergency outside the hospital and is characterized by spontaneous coronary plaque rupture and thrombosis and an associated systemic inflammatory response.^7^ Side-branch occlusion is considered to be the most common cause of type 4a MI in CCS patients undergoing PCI,^53^^,^^54^ but it is likely that its impact on outcome depends on the size of the occluded side branches. The incidence of SBO may be associated with the choice of stent type, but also with the type of procedure [such as chronic total occlusion (CTO), rotational atherectomy, etc.] and the target segment, with the mid-left anterior descending coronary artery having the highest density of side branches.^55–57^ Irreversible myocardial injury due to SBO following PCI can be imaged by CMR as new LGE adjacent to the stent (Role of cardiac imaging section).^48–50^ Distal coronary embolization of intracoronary thrombus and atheromatous material can result in no-reflow/slow-flow during PCI in CCS patients. Embolization may not be preventable, despite current anticoagulant and antiplatelet adjunctive therapy and use of aspiration or protection devices. Irreversible myocardial injury due to coronary embolization following PCI can be imaged by CMR as new LGE downstream of the stent (Role of cardiac imaging section).^48–50^ Thrombosis and neuro-hormonal activation may induce coronary vasospasm during PCI in the epicardial arteries distal to the intervention site and may result in no-reflow/slow-reflow and periprocedural myocardial injury.^58^ Moreover, coronary microcirculatory vasospasm may arise as a consequence of potent vasoconstrictors, such as serotonin and endothelin, released from activated platelets and endothelium.^59^ A neural mechanism of vasoconstriction may also be involved, as α-adrenoreceptor blockade has been shown to attenuate coronary vasoconstriction and increase coronary flow reserve during PCI.^60^ Percutaneous coronary intervention-related factors, such as pre-dilation, partially occlusive devices (such as catheter extension devices, retrograde CTO procedures, atherectomy devices), which are needed for optimal stent placement, can result in prolonged total vessel occlusion times and induce periprocedural myocardial injury. Abrupt vessel closure during PCI is usually caused by dissection proximal or distal to the stent or acute stent thrombosis. Other potential rare periprocedural causes of myocardial injury include coronary artery wire perforation, air embolization, and arrhythmias. Even transient occlusions of the coronary artery during balloon angioplasty inflations have been reported to increase cTn values during PCI in CCS patients.^61^

- In summary, the aetiology of periprocedural myocardial injury and type 4a MI is multifactorial, with SBO and distal embolization being the major causes.

## Independent predictors of major adverse cardiac events following percutaneous coronary intervention

A variety of patient features, lesion characteristics, and periprocedural factors have been shown to be independent predictors of periprocedural myocardial injury, type 4a MI, and MACE, in CCS patients undergoing PCI (*Table 2*, Supplementary material online, *Table S2A and B*).^3^^,^^23–28^^,^^34^^,^^35^^,^^62–68^ Identification of these factors prior to the PCI procedure may help to identify patients at higher risk of experiencing these periprocedural complications and allow the implementation of preventive measures (*Table 3*).^69–88^ Accordingly, these factors should be adjusted for using multivariate logistic regression in studies evaluating the prognostic relevance of post-PCI elevations in cTn. Several studies have shown elevated baseline (pre-PCI) cTn values (present in up to 30% of patients), to be strong independent predictors of MACE in CCS patients undergoing PCI.^28^^,^^34^^,^^35^^,^^67^ This likely reflects a higher risk patient population in terms of patient risk factors, coronary plaque burden, and procedure complexity. Accordingly, studies evaluating whether post-PCI cTn elevation is an independent predictor of MACE should either exclude patients with elevated baseline cTn values or adjust for this factor.

**Table 2:** Independent predictors of periprocedural myocardial injury, type 4a myocardial infarction and major adverse cardiac events in patients undergoing percutaneous coronary intervention

Independent predictors of periprocedural myocardial injury and type 4a MI	Independent predictors of MACE
*Patient factors*	*Patient factors*
Age^3^^,^^62^	Advanced age (≥75 years)^3^^,^^23^^,^^24^^,^^26^^,^^27^^,^^62^^,^^63^
Renal failure^64^^,^^65^	Diabetes^23^^,^^24^^,^^62^
Elevated baseline of cTn^64^	Renal failure^3^
Current congestive heart failure^27^	Peripheral vascular disease^24^
	Previous stroke^27^
*Lesion characteristics*	Previous MI^27^
Multi-vessel^66^	Ever smoked^24^
Bifurcation lesion^64^	COPD^24^
Left main disease^3^^,^^62^	Ejection fraction^24^^,^^63^
	Current congestive heart failure^26^^,^^27^
*Procedure factors*	Elevated baseline of cTn^28^^,^^34^^,^^35^^,^^67^
Stent length^3^^,^^62^^,^^64^	
Stent diameter^62^	*Lesion characteristics*
Number of stents^26^^,^^62^	Left mainstem^3^
Multi-vessel PCI^27^^,^^68^	Calcified^24^^,^^25^
Rotational atherectomy^27^	SVG^24^
Retrograde approach for CTO^68^	
	*Procedure factors*
	Multi-vessel stenting^3^
	Stent length >30 mm^3^
	Post-procedural bleeding^27^

COPD, chronic obstructive pulmonary disease; cTn, cardiac troponin; CTO, chronic total occlusion; MI, myocardial infarction; PCI, percutaneous coronary intervention; SVG, saphenous vein graft.

**Table 3:** Therapeutic strategies for preventing periprocedural myocardial injury and type 4a myocardial infarction in chronic coronary syndrome patients undergoing percutaneous coronary intervention

Agent	Timing of administration	Potential mechanism of action	Main study findings	Strength of evidence
*High-dose Statins*	Pre-PCI	Pleiotropic effect on inflammation^69^^,^^70^ Production of endothelial progenitor cells^70^	↓ Incidence of periprocedural myocardial injury and type 4a MI^71–74^ ↓ Incidence of MACE (death, re-infarction and revascularization)^75^	Multiple randomized controlled trials^71–74^ However, neutral effect in some studies^76–78^
*Cangrelor*	At the time of PCI (intravenous)	Antiplatelet drug	↓ Incidence of periprocedural myocardial injury and type 4a MI^79^	One large randomized controlled trial^79^
*Remote ischaemic conditioning*	Pre-PCI	Reduces acute myocardial ischaemia-reperfusion injury	↓ Incidence of periprocedural myocardial injury and type 4a MI^80–83^ ↓ Incidence of MACE (but not powered for clinical outcomes)^84^	Multiple randomized controlled trials^80–83^ However, neutral effect in one study^85^
*Vitamin C*	Pre-PCI	Antioxidant effects	↑Microcirculatory reperfusion ↓Incidence of periprocedural myocardial injury^86^^,^^87^	Single randomized controlled trial of 532 patients^87^
*Enalaprilat*	At the time of PCI (intracoronary)	Endothelium-dependent epicardial coronary vasodilation mediated by endogenous bradykinin activity	↓ Incidence of periprocedural myocardial injury^88^	Single small randomized controlled trial of 40 patients^88^

MACE, major adverse cardiovascular events; MI, myocardial infarction; PCI, percutaneous coronary intervention.

## Prognostic relevance of periprocedural myocardial injury and type 4a myocardial infarction

Although studies have demonstrated post-PCI elevations of either CK-MB or cTn to be associated with future risk of MACE, cTn (and hs-cTn) have replaced the use of CK-MB at most centres. A number of clinical studies and meta-analyses, but not all, have reported associations between post-PCI elevation of cTn values and increased risk of MACE (Supplementary material online, *Tables*  3A–D). Although several pooled meta-analyses have reported associations between post-PCI elevations of cTn values and clinical outcomes, they did not adjust for factors that are known to impact on the risk of periprocedural myocardial injury, type 4a MI, and MACE^4^^,^^89–91^ (Supplementary material online, *Table S3D*). A recently published large patient-level pooled analysis demonstrated that post-PCI elevations of both CK-MB and cTn values were independently associated with all-cause mortality at 1 year with the following combinations of fold elevations being predictive of outcome: CK-MB ≥5 and cTn ≥35, CK-MB ≥10 and cTn <70, and CK-MB ≥5 and cTn ≥70^23^ (Supplementary material online, *Table S3D*). However, this study did not evaluate whether post-PCI cTn elevation as a continuous variable was predictive of all-cause mortality at 1 year.^23^ Silvain *et al.*^62^ have recently performed a patient-level pooled analysis focused on post-PCI cTn elevations (analysing a different set of studies to that by Garcia-Garcia *et al.*^23^) comprising 9081 CCS patients undergoing PCI (Supplementary material online, *Table S3d*). In this study, care was taken to evaluate the baseline (pre-PCI) cTn value to ensure that the appropriate 99th percentile URL for the assay was used, and if it was not, the study was excluded. The incidence of type 4a MI in a subset of 2316 CCS patients undergoing PCI with normal baseline cTn values was 12.7%, and its occurrence was a strong independent predictor of all-cause mortality at 1 year [adjusted odds ratio (AdjOR) 3.21, 95% confidence interval (1.42–7.27), *P* = 0.005]. These findings confirm the prognostic relevance of the >5× 99th percentile URL cut-off threshold of post-PCI cTn elevation selected by the 4th UDMI for defining type 4a MI. The incidence of periprocedural myocardial injury (defined as post-PCI cTn elevation >1× 99th percentile URL by the 4th UDMI) in CCS patients with normal baseline cTn values was 52.8% (79.8% if the analysis was restricted to hs-cTn), but periprocedural myocardial injury was not associated with all-cause mortality at 1 year (Supplementary material online, *Table S3D*).^62^ These findings suggest that the 4th UDMI definition of periprocedural myocardial injury might be too sensitive, as it is not an independent predictor of all-cause mortality at 1 year. However, the study by Silvain *et al*^.62^ did find that post-PCI cTn elevations >3× 99th percentile URL independently predicted all-cause mortality at 1 year in CCS patients undergoing PCI, suggesting that even relatively low post-PCI elevations of cTn are prognostically relevant (Supplementary material online, *Table S3D*). The analysis may have been underpowered to detect the prognostic relevance of even smaller changes in cTn values. Receiver operating characteristic curve analysis identified a post-PCI cTn cut-off elevation of >5× 99th percentile URL to be the optimum threshold for independently predicting all-cause mortality at 1 year in terms of sensitivity and specificity. Prognostically relevant or ‘major’ periprocedural myocardial injury (defined in this Consensus Document as a post-PCI cTn elevation of >5× 99th percentile URL) occurred in 18.2% of patients with normal baseline cTn values and was an independent predictor of all-cause mortality at 1 year [AdjOR 2.29, 95% CI (1.32–3.97), *P* = 0.004]. Importantly, this post-PCI cTn threshold is identical to that used in the 4th UDMI definition of type 4a MI, simplifying the diagnosis of major periprocedural myocardial injury and type 4a MI. As expected the prognostic implications of type 4a MI are greater than major periprocedural myocardial injury following PCI, with the risk of 1-year all-cause mortality being higher in patients with type 4a MI (AdjOR 3.21) when compared with those patients with major periprocedural myocardial injury (AdjOR 2.29).^62^ These findings confirm that the presence of new ischaemic changes on ECG or angiographic evidence of a flow-limiting complication, as required for type 4a MI, do provide additional prognostic information. In this Consensus Document, we define patients with post-PCI cTn elevations >1× but ≤5× 99th percentile URL as having ‘minor’ periprocedural myocardial injury.

**Figure 2:**
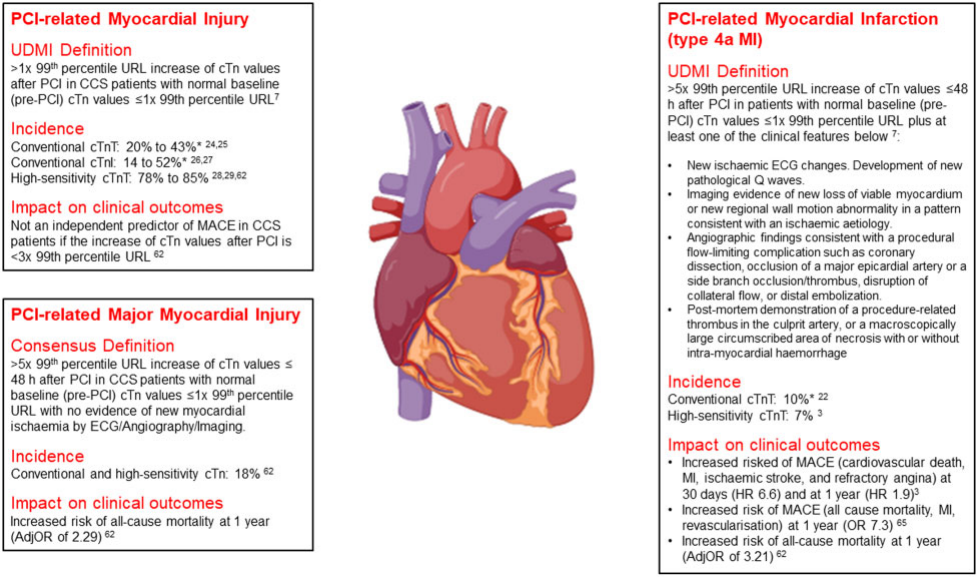
Summary of periprocedural myocardial injury and Type 4a myocardial infarction in chronic coronary syndrome patients undergoing percutaneous coronary intervention. This figure provides an overview of the definitions, incidences, and potential impact on clinical outcomes of periprocedural myocardial injury and type 4a myocardial infarction as defined by the 4th Universal Definition of Myocardial Infarction in chronic coronary syndrome patients undergoing percutaneous coronary intervention. In this Consensus Document, we introduce a new category of major periprocedural myocardial injury, which has been shown to be prognostically relevant in chronic coronary syndrome patients undergoing percutaneous coronary intervention. *Some of these studies included both acute coronary syndrome and chronic coronary syndrome patients. AdjOR, adjusted odds ratio; CCS, chronic coronary syndrome; HR, Hazards Ratio; MACE, major adverse cardiac events; MI, myocardial infarction; OR, odds ratio; PCI, percutaneous coronary intervention; UDMI, Universal Definition of Myocardial Infarction; URL, upper reference limit.

*Figure 2* provides a summary of the definitions, incidence, and impact on clinical outcomes of periprocedural myocardial injury as defined by the 4th UDMI, major periprocedural myocardial injury, and type 4a MI in CCS patients undergoing PCI.

## Management of periprocedural myocardial injury and type 4a myocardial infarction

**Figure 3:**
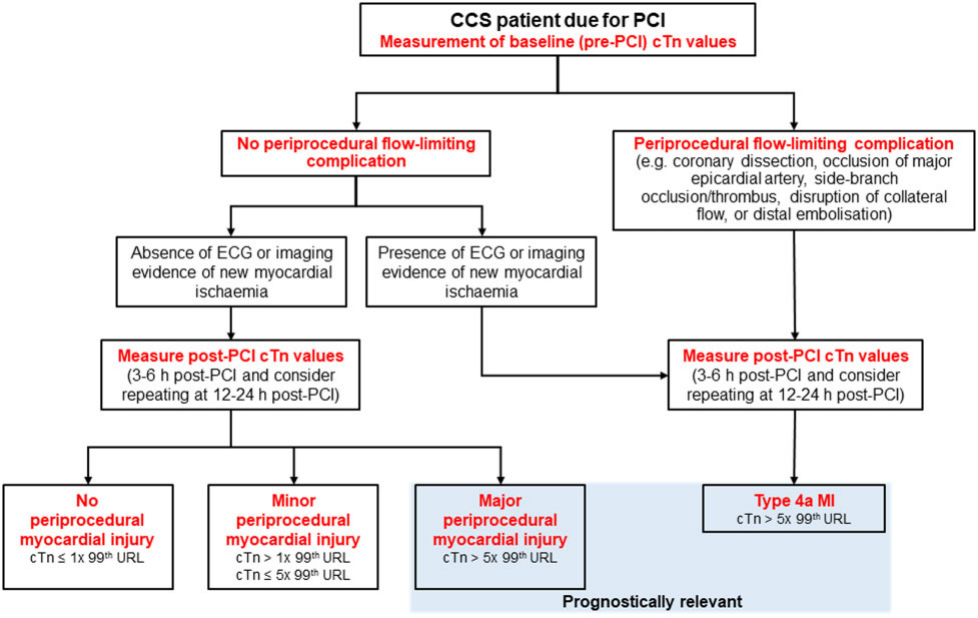
Diagnostic algorithm for periprocedural myocardial injury and type 4a myocardial infarction in chronic coronary syndrome patients undergoing percutaneous coronary intervention. In this Consensus Document, we propose a diagnostic algorithm for periprocedural myocardial injury and type 4a myocardial infarction in chronic coronary syndrome patients undergoing percutaneous coronary intervention, which is based on post-percutaneous coronary intervention elevation of cardiac troponin values, and the presence of ECG/imaging/angiographic evidence of new myocardial ischaemia as stipulated in the 4th Universal Definition of Myocardial Infarction. Patients with suspected major periprocedural myocardial injury, based on post-percutaneous coronary intervention cardiac troponin elevation of >5× 99th percentile URL, the ECG and coronary angiogram should be carefully reviewed, and cardiac imaging (e.g. echocardiography) performed to actively exclude the diagnosis of type 4a myocardial infarction. The presence of either major periprocedural myocardial injury or type 4a myocardial infarction in chronic coronary syndrome patients undergoing percutaneous coronary intervention is prognostically relevant, as both have been shown to be independent predictors of mortality at 1 year post-percutaneous coronary intervention. In patients with elevated baseline (pre-percutaneous coronary intervention) cardiac troponin in whom the cardiac troponin values are stable (≤20% variation) or falling, the post-percutaneous coronary intervention cardiac troponin values must rise by >20%. However, the absolute post-percutaneous coronary intervention value must still be >5× 99th percentile URL to diagnose major periprocedural myocardial injury or type 4a myocardial infarction. CCS, chronic coronary syndrome; PCI, percutaneous coronary intervention; URL, upper reference limit.

Current practice guidelines do not provide specific recommendations for diagnosing and managing periprocedural myocardial injury or type 4a MI in CCS patients undergoing PCI.^2^^,^^92^ Based on a review of current scientific data, and the results of a recent individual-level pooled analysis,^62^ we propose a diagnostic algorithm for periprocedural myocardial injury and type 4a MI in CCS patients with normal (pre-PCI) baseline cTn values undergoing PCI (*Figure 3*). For CCS patients with baseline (pre-PCI) cTn values, which are elevated, stable, or falling, the post-PCI cTn must rise by >20%, and the absolute post-PCI value must still be >5× 99th percentile URL for both major periprocedural myocardial injury and type 4a MI^7^ (*Figure 3*).

### Before the percutaneous coronary intervention procedure

Whether all CCS patients undergoing PCI should undergo routine baseline (pre-PCI) and post-PCI measurements of cTn has been discussed in past guidelines. The ACC/AHA/SCAI 2005 guideline update for PCI^93^ had originally made a class IIa recommendation for routine measurement of cardiac biomarker levels (CK-MB and/or cTn) in all patients undergoing PCI, and at 8–12 h after the procedure, but these recommendations were not included in the ESC/EACTS 2018 guidelines on myocardial revascularization.^92^ In the 4th UDMI, it was recommended that baseline (pre-PCI) and post-PCI cTn values should be routinely measured to detect the occurrence of periprocedural myocardial injury.^7^ In order to make an accurate diagnosis of either major periprocedural myocardial injury or type 4a MI following PCI, prior knowledge of the baseline (pre-PCI) cTn level is required to correctly interpret post-PCI elevations of cTn values.

In this Consensus Document, we recommend that, whenever possible, baseline (pre-PCI) cTn values should be measured in all CCS patients undergoing PCI. For CCS patients undergoing a planned PCI procedure, the blood sample may be undertaken in the cardiac catheterization laboratory from the arterial sheath prior to PCI, and for those CCS patients undergoing initial diagnostic coronary angiography, the blood sample may be taken via the arterial sheath from only those patients proceeding to PCI. It is appreciated that in some centres, routine measurement of baseline (pre-PCI) cTn values may not be possible in all CCS patients undergoing PCI. In this case, one may consider measurement of baseline (pre-PCI) cTn values in only those with patient features, lesion characteristics, and periprocedural factors that have been shown to independently predict major periprocedural myocardial injury, type 4a MI, and MACE following PCI (see *Table 2*).

The 2017 ESC focused update on dual anti-platelet therapy (DAPT) in CAD recommends clopidogrel (600 mg loading dose, 75 mg daily dose) in addition to aspirin in CCS patients undergoing planned PCI (IA recommendation).^94^ This is supported by recent studies in CCS patients undergoing PCI reporting that pre-treatment with the potent platelet P2Y_12_ inhibitors ticagrelor (pre-PCI and daily for 30 days)^95^ or prasugrel (pre-PCI only),^96^ did not reduce periprocedural myocardial injury or MI, with ticagrelor being associated with an increased risk of minor bleeding at 30 days, when compared with clopidogrel. For DAPT-naïve CCS patients who require PCI following diagnostic coronary angiography, it is probably advisable to delay PCI by >2 h or even to the next day, given that a 600 mg loading dose of clopidogrel acts in ∼2 h. However, in those rare instances where *ad-hoc* PCI is urgently required in DAPT-naïve CCS patients, oral loading with soluble aspirin and ticagrelor or crushed prasugrel may be considered given their faster onset of action (30 min), with clopidogrel given thereafter (600 mg loading dose, 75 mg daily dose). In cases of urgent complex *ad-hoc* PCI in DAPT-naïve CCS patients, one may also consider intravenous fast-acting cangrelor to achieve rapid platelet inhibition at time of PCI, based on the results of the CHAMPION PHOENIX trial.^79^

Several other therapeutic strategies have been evaluated for their ability to prevent periprocedural myocardial injury and type 4a MI in CCS patients when given prior to PCI (*Table 3*). Of these, there is substantial evidence to show that high-dose statins (e.g. atorvastatin 80 mg or rosuvastatin 40 mg) administered prior to PCI can reduce the risk of periprocedural myocardial injury, type 4a MI, and MACE in CCS patients.^75^ Low-dose treatment with the anti-inflammatory agent, colchicine, has been reported to reduce mainly ischaemia-driven clinical events in patients with recent MI^97^ and in CCS patients.^98^ However, pre-treatment of CCS with high-dose colchicine prior to PCI failed to reduce the incidence of periprocedural myocardial injury (as defined by the 4th UDMI), type 4a MI, or SCAI-defined periprocedural MI, when compared with placebo.^99^ Whether post-PCI treatment with low-dose colchicine can reduce MACE in CCS patients experiencing type 4a MI post-PCI is not known.

### During the percutaneous coronary intervention procedure

In cases of major intra-procedural vascular complications during PCI (e.g. SBO, dissection, plaque shift, thromboembolism, spasm, or no-reflow/slow-reflow), emergent treatment to restore coronary blood flow is a priority. Intravascular imaging with Intravascular ultrasound (IVUS) or optical coherence tomography should be considered to identify and correct mechanical factors that might contribute to coronary dissection or stent thrombosis.^92^ Glycoprotein IIb/IIIa inhibitors may be considered in specific ‘bail-out’ situations including high intraprocedural thrombus burden, slow-flow, or no-flow with closure of the stented coronary vessel (ESC class IIa level C recommendation).^92^ In cases of vasospasm or no-reflow, the use of intracoronary vasodilators, such as calcium channel blockers, nitroglycerine, nitroprusside, or adenosine, may be helpful, but there are no data to recommend one drug over the other. Chronic coronary syndrome patients with these periprocedural complications will of course be at a greater risk of experiencing periprocedural myocardial injury and type 4a MI and should have post-PCI cTn values measured (*Figure 3*).

### Following the percutaneous coronary intervention procedure

Recurrent ischaemic symptoms post-PCI should prompt immediate ECG assessment and measurement of post-PCI cTn values (class IC recommendation).^93^ Patients with ischaemic symptoms and new ST-segment elevation should be transferred to the cardiac catheterization laboratory without delay. The treatment approach should be individualized according to ECG changes, cTn results, nature and extent of the PCI, technical feasibility, and patient characteristics, when deciding the need for repeat coronary angiography.

In the 4th UDMI, it has been recommended that post-PCI cTn values should be routinely measured to detect the occurrence of periprocedural myocardial injury.^7^ Therefore, in this consensus document, we recommend that, whenever possible, all CCS patients undergoing PCI, should have post-PCI cTn values measured at 3–6 h post-PCI, and where cTn values are rising, further blood sampling may be considered to document the peak cTn value at 12–24 h post-procedure (*Figure 3*).^7^^,^^100^ This is mandatory in those patients who experience periprocedural complications associated with reduced coronary blood flow or have ECG changes indicative of new myocardial ischaemia, so that a diagnosis of type 4a MI can be made. For those patients who are kept in overnight for observation by their treating physician due to periprocedural complications, it may be feasible to measure cTn values at 3–6 h with repeat testing at 12–24 h. However, for those patients with uncomplicated PCI who may be discharged on the same day, the measurement of post-PCI cTn values may only be possible at the 3–6 h time-point. It is appreciated that in some centres, routine measurement of post-PCI cTn values may not be possible in all CCS patients undergoing PCI. In this case, one may consider measurement of post-PCI cTn values in only those with patient features, lesion characteristics, and procedural factors, which have been shown to be independent predictors of major periprocedural myocardial injury, type 4a MI, and MACE following PCI (see *Table 2*).

Chronic coronary syndrome patients diagnosed with type 4a MI following PCI, based on post-PCI cTn elevations of >5× 99th percentile URL within 48 h, and evidence of new myocardial ischaemia (ECG changes or angiography evidence of a flow-limiting complication) should undergo echocardiography or other cardiac imaging to detect the presence of new loss of viable myocardium or new RWMA and assess left ventricular ejection fraction. Chronic coronary syndrome patients diagnosed with type 4a MI are at increased risk of all-cause 1 year of mortality (AdjOR 3.21),^62^ and pharmacotherapy should be optimized to reduce risk of future MACE, as recommended in current ESC revascularization and CCS guidelines.^2^^,^^92^ Whether CCS patients with type 4a MI, who are not already on angiotensin-converting enzyme (ACE) inhibitors (for heart failure, hypertension, or diabetes) or beta-blockers (for left ventricular dysfunction or systolic heart failure), would benefit from the addition of these pharmacotherapies to reduce the risk of future MACE is not known, and needs to be evaluated in future studies.

In the absence of new myocardial ischaemia (new ischaemic changes on ECG or angiographic evidence of a flow-limiting complication), a post-PCI cTn elevation of >5× 99th percentile URL within 48 h post-procedure indicates the occurrence of prognostically relevant major periprocedural myocardial injury (*Figure 3*).^62^ In these patients, a type 4a MI should be actively excluded by careful review of the ECG (for new ischaemic changes) and coronary angiogram (for any subtle periprocedural flow-limiting complication), and an echocardiogram or other cardiac imaging should be performed to exclude an RWMA and to assess left ventricular ejection fraction.^7^ Chronic coronary syndrome patients diagnosed with major periprocedural myocardial injury are at increased risk of 1 year of all-cause mortality (AdjOR 2.29),^62^ and pharmacotherapy should be optimized to reduce risk of future MACE, as recommended in ESC revascularization and CCS guidelines.^2^^,^^92^ Whether CCS patients with prognostically relevant major periprocedural myocardial injury, who are not already on ACE-inhibitors (for heart failure, hypertension, or diabetes) or beta-blockers (for left ventricular dysfunction or systolic heart failure), would benefit from the addition of these pharmacotherapies to reduce the risk of future MACE is not known, and needs to be evaluated in further studies.

Patients with no periprocedural myocardial injury (post-PCI cTn elevation ≤1× 99th percentile URL) or only ‘minor’ periprocedural myocardial injury (post-PCI cTn elevation >1× 99th percentile URL but ≤5× 99th percentile URL) (*Figure 3*) should receive pharmacotherapy, as recommended in current ESC revascularization and CCS guidelines.^2^^,^^92^

## Future research directions

A number of gaps remain in our knowledge with regard to both periprocedural myocardial injury and type 4a MI in patients undergoing PCI, and further research is needed to address this.

- The additional work and costs of routine measurement of baseline (pre-PCI) and post-PCI cTn levels in all CCS patients undergoing PCI are justified because it may provide important registry data and enable future research to confirm the prognostic relevance of cTn levels on clinical outcomes, and inform future recommendations in terms of management strategies and new treatments in CCS patients experiencing major periprocedural myocardial injury and type 4a MI following PCI.
- Further studies are needed to evaluate the prognostic relevance of post-PCI elevations of hs-cTn in CCS patients undergoing PCI, given that the majority of clinical studies have used conventional cTn.
- The optimal time-point(s) for measuring post-PCI cTn values to predict future MACE is not known, and the choice of this timescale will need to recognize that in some centres, CCS patients undergoing uncomplicated PCI are discharged on the same day.
- Clinical studies are needed to validate the diagnostic criteria for defining prognostically relevant major periprocedural myocardial injury and type 4a MI in CCS patients with elevated baseline (pre-PCI) cTn values—this is currently defined in the 4th UDMI as a >20% increase in post-PCI cTn.^7^
- The focus of this Consensus Document has been on the prognostic relevance of periprocedural myocardial injury and type 4a MI in CCS patients undergoing PCI. Further studies are needed to evaluate the prognostic relevance of post-PCI cTn elevations in ACS patients, in whom baseline (pre-PCI) cTn values are elevated and rising.
- Further research is needed to discover novel treatments that can be administered to CCS patients prior to PCI to reduce the risk of periprocedural myocardial injury and type 4a MI. In this regard, the incidence of major periprocedural myocardial injury and type 4a MI may serve as prognostically relevant surrogate clinical endpoints for assessing the efficacy of future cardioprotective therapies.
- Further studies are needed to evaluate new treatments for reducing the future risk of MACE, which can be administered following PCI to patients who experience major periprocedural myocardial injury or type 4a MI. In this regard, whether patients who are not already on ACE-inhibitors (for heart failure, hypertension, or diabetes) or beta-blockers (for left ventricular dysfunction or systolic heart failure), would benefit from the addition of these pharmacotherapies to reduce the risk of future MACE is not known and remains to be tested.
- Periprocedural MI has been used as a primary composite endpoint in recent clinical trials of CCS patients undergoing PCI,^12–15^ and the choice of periprocedural MI definition (protocol-specific vs. type 4a MI vs. SCAI) has been shown to impact on the outcomes of these trials.^12–15^^,^^101^ As such, further research is needed to better define periprocedural MI when used as a primary composite endpoint in clinical trials. We would recommend that the type 4a MI definition is used in this situation, given its known prognostic impact on mortality.^3^^,^^62^ Whether modest isolated post-PCI elevations of cTn >5× 99th percentile URL (indicative of periprocedural major myocardial injury) should be used as a primary composite endpoint as well needs to be evaluated in future research studies.^101^
- The digital innovation in healthcare has increased the quantity and quality of patient-generated health data. Machine learning algorithms have been used to enhance risk prediction of post-PCI acute kidney injury,^102^ bleeding,^103^^,^^104^ and clinical outcomes,^105^ thereby improving clinical decision-making before and during PCI in CCS patients. Whether they can also be used to improve risk prediction of post-PCI complications such as periprocedural myocardial injury and type 4a MI is not known, and warrants further investigation.

## Consensus recommendations

- *Baseline (pre-PCI) cTn values:* Baseline (pre-PCI) cTn values should be measured, whenever possible, in all CCS patients undergoing PCI, as knowledge of this information is essential to correctly interpret post-PCI elevations in cTn values,^28^^,^^34^^,^^35^^,^^67^ and to diagnose major periprocedural myocardial injury and type 4a MI following PCI (*Figure 3*;  *Graphical abstract*).
- *Post-PCI cTn values:* Post-PCI cTn values should be measured, whenever possible, at 3–6 h post-procedure, and if the values are rising, further sampling may be considered at 12–24 h post-procedure in all CCS patients undergoing PCI. For those with concurrent ECG, imaging or angiographic evidence of new myocardial ischaemia, the diagnosis of type 4a MI may apply (*Figure 3*).^7^ For those without concurrent ECG, imaging or angiographic evidence of new myocardial ischaemia, the diagnosis of major periprocedural myocardial injury may apply (*Figure 3*).^62^
- *Type 4a MI:* In CCS patients with normal baseline (pre-PCI) cTn values (≤1× 99th percentile URL) or elevated but stable baseline cTn values undergoing PCI who experience a type 4a MI, pharmacotherapy should be optimized to reduce risk of future MACE as recommended in current ESC revascularization and CCS guidelines.^2^^,^^92^ Whether CCS patients with type 4a MI, who are not already on ACE-inhibitors (for heart failure, hypertension, or diabetes) or beta-blockers (for left ventricular dysfunction or systolic heart failure), would benefit from the addition of these drugs for reducing the risk of future MACE is not known, and needs to be evaluated in time to come studies. As type 4a MI is a strong independent predictor of all-cause mortality at 1 year post-PCI, its incidence may be used as a quality metric and surrogate endpoint for clinical trials.^3^^,^^62^^,^^65^
- *Major periprocedural myocardial injury:* In CCS patients with normal baseline cTn values (≤1× 99th percentile URL) or elevated but stable baseline cTn values undergoing PCI who experience prognostically relevant major periprocedural myocardial injury, defined as post-PCI cTn elevation >5× 99th percentile URL (in the absence of ECG, angiographic, and imaging evidence of new myocardial ischaemia) within 48 h of PCI,^62^ pharmacotherapy should be optimized to reduce risk of future MACE as recommended in current ESC revascularization and CCS guidelines.^2^^,^^92^ Whether CCS patients with prognostically relevant major periprocedural myocardial injury, who are not already on ACE-inhibitors (for heart failure, hypertension, or diabetes) or beta-blockers (for left ventricular dysfunction or systolic heart failure), would benefit from the addition of beta-blockers or ACE-inhibitors to reduce the risk of future MACE is not known, and needs to be further evaluated. As major periprocedural myocardial injury is an independent predictor of all-cause mortality at 1 year,^62^ its incidence may be used as a quality metric and surrogate endpoint for clinical trials (*Figure 3*).
- *‘Minor’ periprocedural myocardial injury:* Chronic coronary syndrome patients with normal baseline cTn values (≤1× 99th percentile URL) undergoing PCI who experience ‘minor’ periprocedural myocardial injury, defined as post-PCI cTn elevation of >1× 99th percentile URL but ≤5× 99th percentile URL (*Figure 3*),^62^ pharmacotherapy should be optimized to reduce risk of future MACE as recommended in current ESC revascularization and CCS guidelines.^2^^,^^92^
- Future clinical studies and meta-analyses evaluating the prognostic relevance of post-PCI elevations in cTn should only include CCS patients with normal baseline (pre-PCI) cTn values (≤1× 99th percentile URL), and should adjust for known patient features, lesion characteristics, and periprocedural factors, which have been shown to be independent predictors of periprocedural myocardial injury, type 4a MI, and MACE (*Table 2*).

## Summary

The prognostic relevance of post-PCI elevations in cardiac biomarkers in CCS patients undergoing PCI has long been debated in the literature, and due to lack of scientific data, the cut-off thresholds of post-PCI cTn elevation used for defining periprocedural myocardial injury and infarction, have been selected based on consensus expert opinions.^5–7^ With respect to type 4a MI in CCS patients with normal baseline (pre-PCI) cTn or elevated but stable baseline cTn values, published studies^3^^,^^65^ and a recent patient-level pooled analysis^62^ have validated the post-PCI cut-off threshold of cTn >5× 99th percentile URL, and have shown it to be a strong independent predictor of all-cause mortality at 1 year. The major issue has been with periprocedural myocardial injury, which has been defined by the 4th UDMI, as any post-PCI elevation in cTn >1× 99th percentile URL in CCS patients with normal baseline (pre-PCI) cTn values. This cut-off value for post-PCI cTn elevation might be too low given that up to 80% (using hs-cTn) of patients experience periprocedural myocardial injury according to this definition, and the fact that its occurrence does not independently predict all-cause mortality at 1 year.^62^ However, a post-PCI elevation in cTn of >3× 99th percentile URL was found to be independently associated with an increased risk of all-cause mortality at 1 year, suggesting that even relatively low levels of post-PCI cTn elevation are prognostically relevant. It has been reported that the optimum threshold for independently predicting all-cause mortality at 1 year post-PCI in CCS patients with normal baseline (pre-PCI) cTn values was >5× 99th percentile URL.^62^ Therefore, in this Consensus Document, we have defined this cut-off to signify the occurrence of prognostically relevant major periprocedural myocardial injury. Importantly, this cut-off threshold is identical to that used to define type 4a MI, thereby simplifying the diagnoses of major periprocedural myocardial injury and type 4a MI. In this Consensus Document, we present a diagnostic algorithm for minor and major periprocedural myocardial injury and type 4a MI in CCS patients undergoing PCI, based on post-PCI cTn values and ECG/imaging/angiographic evidence of new myocardial ischaemia (*Figure 3*). Further research is needed to evaluate novel treatments for reducing the risk of type 4a MI and major periprocedural myocardial injury in CCS patients undergoing PCI, and further studies are needed to evaluate pharmacotherapies for reducing the risk of future MACE in those CCS patients who experience these PCI-related complications.

## Supplementary material

Supplementary material is available at *European Heart Journal* online.

## Data availability

No new data were generated or analysed in support of this research.

## Supplementary Material

ehab271_Supplementary_Tables
