# Supplementary material for: Prognostically relevant periprocedural myocardial injury and infarction associated with percutaneous coronary interventions: a Consensus Document of the ESC Working Group on Cellular Biology of the Heart and European Association of Percutaneous Cardiovascular Interventions (EAPCI)
Source: Eur Heart J. 2021 May 31;42(27):2630–42. doi: 10.1093/eurheartj/ehab271 (PMC8282317; doi:10.1093/eurheartj/ehab271)
Supplement: ehab271_Supplementary_Tables [file ehab271_supplementary_tables.docx]

**Supplementary Table 1**

**Definitions of procedural myocardial injury and infarction in patients with elevated baseline (pre-PCI) cTn levels**

| **Definition** | **Myocardial injury** | **Myocardial infarction** |
| --- | --- | --- |
| Second UDMI (2007)^1^ | Not available | Type 4a MI  If cardiac troponin is elevated before the procedure and not stable for at least two samples 6 h apart, there are insufficient data to recommend biomarker criteria for the diagnosis of peri-procedural myocardial infarction.  If the values are stable or falling, criteria for reinfarction by further measurement of biomarkers together with the features of the ECG or imaging can be applied. |
| Third UDMI (2012)^2^ | Not available | Type 4a MI  In patients with elevated pre-procedure cTn in whom the cTn levels are stable (≤20% variation) or falling, the post-procedure cTn must rise by >20%. However, the absolute post-procedural value must still be at least 5x 99th percentile URL |
| SCAI (2014)^3^ | Not available | Clinically relevant MI  In patients with elevated baseline cTn in whom the biomarker levels are stable or falling: The cTn rises by an absolute increment equal to those levels recommended in Supplementary Table 1a from the most recent pre-procedure level.  In patients with elevated cTn in whom the biomarker levels have not been shown to be stable or falling: The cTn rises by an absolute increment equal to those levels recommended in Supplementary Table 1a plus new ST-segment elevation or depression plus signs consistent with a clinically relevant MI, such as new onset or worsening heart failure or sustained hypotension. |
| ARC-2 (2018)^4^ | Not available | Myocardial Infarction  ≥35x URL cTn increase with patients with normal plus at least one of the following   1. New significant Q waves or equivalent 2. Flow-limiting angiographic complications in a major epicardial vessel or >1.5-mm diameter branch 3. New “substantial” loss of myocardium on imaging |
| Fourth UDMI (2018)^5^ | Cardiac procedural myocardial  Injury  A rise of cTn values >20% of the baseline value when it is above the 1x 99th percentile URL, but it is stable or falling. | Type 4a MI  In patients with elevated pre-procedure cTn in whom the cTn levels are stable (≤20% variation) or falling, the post-procedure cTn must rise by >20%. However, the absolute post-procedural value must still be at least 5x 99^th^ percentile URL |

**Supplementary Table 2a**

**Independent predictors of procedural myocardial injury and type 4a MI in patients undergoing PCI**

| **Independent predictor** | **Increase in risk** | **References** |
| --- | --- | --- |
| **Patient factors** | | |
| Age | OR 2.2 >75 years old  AdjOR 1.07 (per 5 years’ increase in age) | Zeitouni et al 2008^6^  Silvain et al 2020^7^ |
| Renal failure | OR 0.81 per 20 ml/min GFR  OR 3.0 | Koskinas et al 2018^8^  Liou et al 2015^9^ |
| Elevated baseline cTn | OR 1.43 | Koskinas et al 2018^8^ |
| Current congestive heart failure | HR 1.49 | Feldman et al 2009^10^ |
| **Lesion characteristics** | | |
| Multi-vessel | OR 1.65 | Chen et al 2017^11^ |
| Bifurcation lesion | OR 1.49 | Koskinas et al 2018^8^ |
| Left main disease | OR 2.2  AdjOR 1.19 | Zeitouni et al 2008^6^  Silvain et al 2020^7^ |
| Rentrop collateral grade ≥2 for CTO | OR 0.27 | Di Serafino et al 2016^12^ |
| **Procedure factors** | | |
| Stent length | OR 1.32 per 10mm stent length  OR 2.0 stent length >30mm  AdjOR 1.13 per 10mm increase | Koskinas et al 2018^8^  Zeitouni et al 2008^6^  Silvain et al 2020^7^ |
| Stent diameter | AdjOR 1.02 per 0.25 mm increase) | Silvain et al 2020^7^ |
| Number of stents | OR 1.5 ≥ 3 stents | Zeitouni et al 2008^6^  Loeb et al 2010^13^ |
| Multi-vessel PCI | HR 1.97  OR 1.91 | Feldman et al 2009^10^  Di Serafino et al 2016^12^ |
| Rotational atherectomy | HR 4.14 | Feldman et al 2009^10^ |
| Retrograde approach for CTO | OR 5.06 | Di Serafino et al 2016^12^ |

**Supplementary Table 2b**

**Independent predictors of MACE in patients undergoing PCI**

| **Independent predictor** | **Increase in risk** | **References** |
| --- | --- | --- |
| **Patient factors** | | |
| Advanced age (≥75 years) | HR 1.06 ≥75 years  HR 1.05 per year  HR 1.39  >75 years  AdjOR 1.45 (per 5 years’ increase in age) | Auguadro et al 2015^14^  Feldman et al 2009^10^  Loeb et al 2010^13^  Prasad et al 2006^15^  Zeitouni et al 2018^6^  Garcia Garcia et al 2019^16^  Silvain et al 2020^7^ |
| Diabetes | HR 1.69  HR 1.46  AdjOR 1.86 | Prasad et al 2006^15^  Garcia Garcia et al 2019^16^  Silvain et al 2020^7^ |
| Hyperlipidaemia | HR 0.66 | Garcia Garcia et al 2019^16^ |
| Renal failure | GFR<60 ml/min | Zeitouni et al 2018^6^ |
| Peripheral vascular disease | HR 1.72 | Prasad et al 2006^15^ |
| Previous stroke | HR 2.09 | Feldman et al 2009^10^ |
| Previous MI | HR 1.95 | Garcia Garcia et al 2019^16^ |
| Ever smoked | HR 1.58  AdjOR 1.69 | Prasad et al 2006^15^  Silvain et al 2020^7^ |
| COPD | HR 2.29 | Feldman et al 2009^10^  Loeb et al 2010^13^ |
| Ejection fraction | HR 0.97 EF<50%  HR 2.5 | Auguadro et al 2015^14^  Prasad et al 2006^15^ |
| Current congestive heart failure | HR 5.0 | Feldman et al 2009^10^  Loeb et al 2010^13^ |
| Hb level | HR 0.82 per g/dl | Feldman et al 2009^10^ |
| Elevated baseline cTn |  | Ndrepapa et al 2016^17^  Miller et al 2006^18^  Prasad et al 2008^19^  Jeremias et al 2008^20^ |
| **Lesion characteristics** | | |
| Left mainstem |  | Zeitouni et al 2018^6^ |
| Calcified | HR 2.03  HR 1.24 | Prasad et al 2006^15^  Herrmann et al 2014^21^ |
| SVG | HR 1.99 | Prasad et al 2006^15^ |
| **Procedure factors** | | |
| Multi-vessel stenting |  | Zeitouni et al 2018^6^ |
| Stent length >30mm |  | Zeitouni et al 2018^6^ |
| Glycoprotein IIb/IIIa | HR 0.45  HR 0.58 | Feldman et al 2009^10^  Prasad et al 2006^15^ |
| Post-procedural bleeding | HR 2.64 | Feldman et al 2009^10^ |

**Supplementary Table 3a**

**Summary of recent major studies evaluating the prognostic relevance of post-PCI cTnT elevations in patients undergoing planned PCI**

| **Reference and study design** | **Patients** | **Normal troponin levels at baseline (pre-PCI)** | **Multivariate adjustment for predictors** | **Timing of post-PCI cTn tests** | **Threshold of post-PCI cTn elevation tested** | **Main results** | **Notes** |
| --- | --- | --- | --- | --- | --- | --- | --- |
| Prasad et al 2006^15^  Single-centre | 1,949 CCS and ACS patients (2000 to 2003) | Mixed | Age  Diabetes  PAD  LVEF <40%  Calcified lesion  SVG  Smoking  GP IIb/IIIa | 8 and 16 h | cTnT > 10% coefficient of variation value  (incidence 19.6%) | ***Independent predictor*** for increased risk of all cause death at 2.2 years (HR 1.2). | Study only included patients with post-PCI CK-MB <99^th^ percentile URL |
| Novack et al 2012^22^  Multi-centre  (60 sites in US) | 4,930 CCS patients (2004 to 2007) | Yes | Age  Sex  Diabetes  CHF  Previous MI  Previous CABG  CKD  Lesion location  N diseases vessel  N treated vessel  Max balloon Diameter  Total stent length | 6 and 24 h | cTnT >3x ULN  (incidence 24.3%)  cTnT >20x ULN (incidence 7%) | ***Independent predictor*** of mortality at 1 year (HR 1.35)  Optimal threshold for ***independent predictor*** of 1 year mortality (HR 2.6) |  |
| Herrmann et al 2014^21^  Single-centre | 5,268 CCS and ACS patients (2000 to 2009) | Yes | Mayo long-term mortality risk score*  Calcified lesion  VSG  Type C lesion  Post-PCI TIMI flow 0/I/II  Urgent PCI  Bifurcation lesion  Thrombotic lesion  Branch occlusion  N of stents  Coronary embolus | 8 and 16 h | cTnT >1x ULN (incidence 43%)  cTnT >25x ULN (incidence 7%) | ***Independent predictor*** of mortality at 3 mths (HR 1.24 per doubling of cTnT).  Weak ***Independent predictor*** of mortality at 67 mths (HR 1.03 per doubling of cTnT).  Optimal threshold for predicting 3 mth mortality was >25 × ULN, which was an ***independent predictor*** of mortality at 3 mths (HR 4.53) | Small number of events at 3 mths. |
| Christensen et al 2016^23^  Single-centre | 2,760 CCS patients (2000 to 2012) | Yes | Age  Gender | 3 and 24 h | cTnT >5x URL (incidence 15%) | Not associated with increased risk of mortality at 5.8 years |  |
| Di Serafino et al 2016^12^  Three-centres | 715 stable CTO patients  (2006 to 2012) | Mixed | No | 12 and 24 h | cTnT >5x 99^th^ percentile URL (incidence 15%) | Not associated with increased risk of MACE (death, MI, revascularisation) at 2 years | Only CTO |
| Yang et al 2017^24^  Single-centre retrospective study | 516 CCS and ACS CAD and NSTEMI patients  (Jan 2013 to Dec 2013) | Mixed | No | 6 and 24 h | cTnT <5x 99^th^ percentile URL for (incidence 22.5%)  cTnT >5x 99^th^ percentile URL (Incidence of Third UDMI type 4a MI (incidence 10.3%) | Associated with increased rates of combined endpoint of death and reinfarction (11% vs 3% in control).  Associated with increased rates of combined endpoint of death and reinfarction (12% vs 3% in control. | Small study with low number of events. |
| Goliasch et al 2019^25^  Single-centre | 2,362 stable CTO patients | Yes | Age  Successful PCI  intervention approach  3-vessel disease Previous CABG Creatinine  Reduced LVEF | 6, 8 and 24 h | cTnT >18x URL (incidence 20%) | Optimal threshold was ***independent predictor*** of all-cause death (HR 1.56) and MACE (death, MI, revascularisation) (HR 1.52) at median follow-up of 2 years | Only CTO |

PAD: peripheral artery disease, LVEF: left ventricle ejection fraction; SVG: saphenous vein graft; GP: glycoprotein; CHF: Congestive heart failure; CKD: Chronic kidney disease; PCI: percutaneous coronary intervention; CABG: coronary artery bypass graft

*Based on age, history of congestive heart failure, body mass index, ejection fraction, MI 1 to 7 days before PCI, pre-PCI troponin elevation (which were absent in our population), former smoker, previous coronary artery bypass graft, history of high cholesterol, glycoprotein IIb/IIIa use, a score based on the use of 4 medications at discharge (aspirin, β-blockers, angiotensin-converting enzyme inhibitors, and lipid-lowering drugs), and a comorbidity index based on 10 risk factors (current smoker, hypertension, history of cerebrovascular/transient ischemic attack, diabetes mellitus, diabetes mellitus with sequelae), chronic obstructive pulmonary disease, peripheral vascular disease, tumor, metastatic cancer, and moderate/severe renal disease).

**Supplementary Table 3b**

**Summary of recent major studies evaluating the prognostic relevance of post-PCI cTnI elevations in patients undergoing planned PCI**

| **Reference and study design** | **Patients** | **Normal troponin levels at baseline (pre-PCI)** | **Multivariate adjustment for predictors** | **Timing of post-PCI cTn tests** | **Threshold of post-PCI cTn elevation tested** | **Main results** | **Notes** |
| --- | --- | --- | --- | --- | --- | --- | --- |
| Feldman et al 2009^10^  Single-centre | 1,601 CCS and ACS patients (2004 to 2005) | Yes | Current CHF  Age  Post PCI bleeding  Previous stroke  COPD  GP IIb/IIIa  Haemoglobin level | 8,16 and 24 h | cTnI >1x 99^th^ percentile URL (incidence 51.9%) | ***Independent predictor*** for increased risk of all cause death at 2 years (HR 1.62). | Study only included patients with no post-PCI elevation in CK-MB. |
| Labriolle et al 2009^26^  Single-centre | 3,200 CCS patients (2003 to 2006) | Yes | No | 6-8 and 12-24 h | cTnI >97.5^th^ percentile URL (incidence 43.8%)  cTnI >3x 99^th^ percentile URL (incidence 23.4%) | No association with MACE (death, reinfarction or revascularisation) at 1 year  No association with MACE (death, reinfarction or revascularisation) at 1 year | Study excluded patients with clinical evidence of ischaemia. |
| Cavallini et al 2010^27^  Multi-centre  (16 sites Italy) | 2,362 CCS and ACS patients (2004 to 2005) | Mixed | SBO  Trans abrupt vessel closure  Distal thromboembolism Transient slow flow | 12 and 24 h | cTnI >1x URL (incidence 39.4%)  cTnI >3x URL (incidence 19.7%) | No association with increased mortality at 2 years  Associated with increased mortality at 2 years (HR 1.68) but this was not significant after adjustment. | Study only included patients with post-PCI CK-MB <99^th^ percentile URL |
| Loeb et al 2010^13^  Single-centre | 907 CCS patients (1998 to 2006) | Yes | Age  Diabetes  COPD  PAD  CHF  Renal failure  N treated lesions  N of stents used  SVG  DES  Rotablation Balloon only | 12 h | cTnI >1.5 ng/ml (ULN)  (incidence 13.7%) | ***Independent predictor*** for MACE (mortality, hospitalisation for AMI and/or unstable angina) |  |
| Jaguszewski et al 2011^28^  Single-centre | 1,110 CCS CTO patients (2003 to 2011) | Yes | Age  Procedural success  Location of lesion treated | 12 and 24 h | cTnI >5x 99^th^ percentile URL (incidence 4.7%) | Associated with increased mortality at 1 year (HR 5.72) and MACCE (death, MI, revascularization, stroke) at 1 year but these was not significant after adjustment. | Study only included CTO patients |
| Auguadro et al 2015^14^  Single-centre | 1,532 CCS patients (2004 to 2010) | Yes | Age  LVEF | 12, 18, 24, 36 and 48 h | cTnI >1.0 ng/ml (incidence 17%) | ***Independent predictor*** for MACE (mortality, hospitalisation for AMI and/or unstable angina) at mean follow-up of 2.2 years (HR 1.03) | Study only included patients with post-PCI CK-MB <99^th^ percentile URL |
| Ferreira et al 2017^29^  Single-centre | 407 CCS patients (2013 to 2014) | Yes | Age  Gender  Hypertension  Diabetes  Hyperlipidemia  CHF  Prior MI or CAD  Haemoglobin  Creatinine  Pre-PCI medications  Multivessel disease  Multivessel PCI  Multistenting | 6 and 24 h | cTnI >1x 99^th^ percentile URL (incidence 74.7%)  cTnI >5x 99^th^ percentile URL (incidence 41.3%) | Associated with increased mortality at 1 year (6.6% vs 1.1%)  Associated with increased mortality at 1 year (8.1% vs 3.1%) | Small study with low number of events. |

CHF: Congestive heart failure; COPD: Chronic obstructive pulmonary disease; PCI: percutaneous coronary intervention; GP: Glycoprotein; SBO: side branch occlusion; PAD: peripheral artery disease; SVG: Saphenous vein graft; DES: drug eluting stent; LVEF: left ventricle ejection fraction; MI: myocardial infarction; CAD: coronary artery disease

**Supplementary Table 3c**

**Summary of recent major studies evaluating the prognostic relevance of post-PCI hs-cTnT elevations in patients undergoing planned PCI**

| **Reference and study design** | **Patients** | **Normal troponin levels at baseline (pre-PCI)** | **Multivariate adjustment for predictors** | **Timing of post-PCI cTn tests** | **Threshold of post-PCI cTn elevation tested** | **Main results** | **Notes** |
| --- | --- | --- | --- | --- | --- | --- | --- |
| Liou et al 2015^9^  Single-centre | 459 CCS and ACS patients (2012 and 2013) | Mixed | CKD  Family history  GP IIb/IIIa  Clinical indication  Lesion complexity  PCI complications Antithrombotic drug | 12 and 48 h | Isolated Hs-cTnT >5x URL (incidence 28.5%)  Hs-cTnT >5x 99^th^ percentile URL (incidence of Third UDMI type 4a MI 4.4%) | ***Independent predictor*** for MACE (all cause mortality, MI, revascularisation) at 1 year (OR 7.3) | Small study with low number of events. |
| Ndrepepa et al 2016^17^  Single-centre | 5,626 CCS and ACS patients (2009 to 2015) | Mixed  3,463 normal  2,163 elevated | Diabetes  Multivessel disease  Hypertension  Baseline troponin  Previous MI  Restenotic lesion  B2/C lesion  GFR  BMI  Bifurcation lesion  LAD intervention  Max balloon pressure  Post-PCI TIMI flow  Total stented length | 6 and 48 h | Hs-cTnT >1x 99^th^ percentile URL (incidence 78%) | Associated with increased mortality at 3 years (HR 2.38) but this was not significant after adjustment. | Baseline hs-cTnT levels an independent predictor (HR 1.22) |
| Koskinas et al 2018^8^  Two-centre | 8,140 CCS patients (2009 to 2015) | Mixed  3,217 normal  4,923 elevated | Bifurcation lesion  PrePCI hs-cTnT  Total stented length  GFR | 6, 24 and 48 h | Hs-cTnT >70x URL (incidence of SCAI PCI-related MI 1.7%)  Hs-cTnT >10x URL- optimal threshold for predicting mortality (incidence 14.6%) | SCAI-defined PCI-related MI independent, highly  specific (98.4%), but insensitive (8.2%) ***independent predictor*** of 1-year mortality (HR 4.1)  Optimal trade-off between sensitivity (85.7%) and specificity (25.4%) was observed at hs-cTnT >10x 99^th^ percentile URL. ***Independent predictor*** of 1-year mortality (HR 1.7) | Clinical evidence of ischaemia not assessed |
| Cottens et al 2018^30^  Two-centre | 411 stable CTO patients  (2011 to 2015) | No | No | 12-24 h | Hs-cTnI >1x 99^th^ percentile URL (incidence 85%)  Hs-cTnT >5x 99^th^ percentile URL (incidence 42%) | Not associated with increased risk of MACCE (death, MI, revascularisation, stroke) at 1 year  Not associated with increased risk of MACCE (death, MI, revascularisation, stroke) at 1 year | Only CTO |
| Zeitouni et al 2018^6^  Single-centre | 1,390 CCS patients (2014 and 2015) | Yes | Diabetes  BMI  LVEF  Left main disease  Stent length > 30 mm  Age  GFR  Number of stents > 3  Multivessel disease | 48 h | Hs-cTnT increase  >5x 99th percentile URL  (incidence of Third UDMI myocardial injury 21.6%)  Hs-cTnT >5x 99^th^ percentile URL for Third UDMI type 4 a MI (incidence 7%) | Myocardial injury was an ***independent predictor*** of ischemic events (cardiovascular death, MI, ischaemic stroke, and refractory angina) at 30 days (HR 4.4) but not at 1 year.  Type 4a MI was an ***independent predictor*** of ischemic events at 30 days (HR 6.6) and 1 year (HR 1.9). |  |

CKD: Chronic kidney disease; GP: Glycoprotein; PCI: percutaneous coronary intervention; GFR: Glomerular filtration rate; BMI: Body mass index; LAD: Left anterior descending artery; LVEF: Left ventricle ejection fraction, BMI: body mass index

**Supplementary Table 3d**

**Summary of recent meta-analyses and pooled analyses investigating the prognostic relevance of post-PCI cTn elevations in patients undergoing planned PCI**

| **Reference and study design** | **Patients** | **Normal troponin levels at baseline (pre-PCI)** | **Multivariate adjustment for predictors** | **Threshold of post-PCI cTn elevation tested** | **Main results** | **Notes** |
| --- | --- | --- | --- | --- | --- | --- |
| Testa et al 2009^31^  Pooled meta-analysis | 7,578 CCS and ACS patients  15 studies | Mixed | No | Mixed cut-off levels of cTnT and cTnI for myocardial injury (incidence 29%)  cTn >3x 99^th^ percentile URL for Second UDMI myocardial infarction (incidence 15%) | Associated with increased risk of MACE (all-cause death, MI, repeat target vessel PCI and CABG) at 18 months (OR 1.48).  Associated with increased risk of MACE at 18 months (OR 2.25) |  |
| Nienhuis et al 2009^32^  Pooled meta-analysis | 15,581 CCS and ACS patients  20 studies (1998 and 2007) | Mixed | No | Mixed cut-off levels of cTnT and cTnI for myocardial injury (incidence 33%) | Associated with increased risk of all-cause death at mean follow-up of 16 months (OR 1.35) |  |
| Feldman et al 2011^33^  Pooled meta-analysis | 22,353 CCS and ACS patients  15 prospective and 7 retrospective  registry/cohort studies (1998 and 2009) | Mixed | No | Mixed cut-off levels of cTnT and cTnI for myocardial injury  (incidence 32%) | Associated with increased risk of all-cause mortality at mean follow-up of 18 months (OR 1.45) |  |
| Garcia Garcia et al 2019^16^  Patient-level meta-analysis | 10,639 CCS patients  4 prospective study and 1 registry  (2008 and 2014) | Yes | Age  Prior MI  Lesion complexity, Hyperlipidaemia, Diabetes | cTn >1x ULN for myocardial injury (incidence 68.4%)  cTn ≥35x ULN  (SCAI definition of PCI-related MI incidence 2.93%) | Not associated with increased mortality at 1 year.  Associated with increased mortality at 1 year but this was not significant after adjustment. | CK-MB elevation ≥10 x 99^th^ percentile URL independent predictor of 1 year mortality |
| Li et al 2019^34^  Pooled meta-analysis | 44,972 CCS patients  24 prospective studies  (1999-2018) | No | No | cTnI >3x 99^th^ percentile URL for myocardial injury  cTnI >3x 99^th^ percentile URL for myocardial injury | Associated with increased mortality at 1 year (OR 1.42)  Associated with increased mortality at 1 year (OR 1.51) | CK-MB elevation ≥1 x 99^th^ percentile URL associated with increased 1 year mortality |
| Silvain et al 2020^7^  Patient-level pooled analysis | 9,081 CCS patients  prospective studies | Yes | Baseline clinical and angiographic variables | cTn >1x 99^th^ percentile URL for procedural myocardial injury  (incidence 52.8% with standard cTn, and 79.8% with hs-cTn)  cTn >5x 99^th^ percentile URL for major procedural myocardial injury  (incidence 18.2%)  cTn >5x 99^th^ percentile URL with new evidence of myocardial ischaemia  (Fourth UDMI of type 4a MI PCI-related MI incidence 12.7%) | Not associated with increased mortality at 1 year.  Major procedural myocardial injury was an ***independent predictor*** of death at 1 year (AdjOR 2.29).  Type 4a MI was an ***independent predictor*** of death at 1 year (AdjOR 3.21). |  |

**Reference List**

1. Thygesen K, Alpert JS, White HD. Universal definition of myocardial infarction. *Eur Heart J*. 2007;**28**:2525-2538.

2. Thygesen K, Alpert JS, Jaffe AS, Simoons ML, Chaitman BR, White HD, Thygesen K, Alpert JS, White HD, Jaffe AS, Katus HA, Apple FS, Lindahl B, Morrow DA, Chaitman BA, Clemmensen PM, Johanson P, Hod H, Underwood R, Bax JJ, Bonow RO, Pinto F, Gibbons RJ, Fox KA, Atar D, Newby LK, Galvani M, Hamm CW, Uretsky BF, Steg PG, Wijns W, Bassand JP, Menasche P, Ravkilde J, Ohman EM, Antman EM, Wallentin LC, Armstrong PW, Simoons ML, Januzzi JL, Nieminen MS, Gheorghiade M, Filippatos G, Luepker RV, Fortmann SP, Rosamond WD, Levy D, Wood D, Smith SC, Hu D, Lopez-Sendon JL, Robertson RM, Weaver D, Tendera M, Bove AA, Parkhomenko AN, Vasilieva EJ, Mendis S. Third universal definition of myocardial infarction. *Eur Heart J*. 2012;**33**:2551-2567.

3. Moussa ID, Klein LW, Shah B, Mehran R, Mack MJ, Brilakis ES, Reilly JP, Zoghbi G, Holper E, Stone GW. Consideration of a new definition of clinically relevant myocardial infarction after coronary revascularization: an expert consensus document from the Society for Cardiovascular Angiography and Interventions (SCAI). *Catheter Cardiovasc Interv*. 2014;**83**:27-36.

4. Garcia-Garcia HM, McFadden EP, Farb A, Mehran R, Stone GW, Spertus J, Onuma Y, Morel MA, van Es GA, Zuckerman B, Fearon WF, Taggart D, Kappetein AP, Krucoff MW, Vranckx P, Windecker S, Cutlip D, Serruys PW. Standardized End Point Definitions for Coronary Intervention Trials: The Academic Research Consortium-2 Consensus Document. *Eur Heart J*. 2018;**39**:2192-2207.

5. Thygesen K, Alpert JS, Jaffe AS, Chaitman BR, Bax JJ, Morrow DA, White HD. Fourth universal definition of myocardial infarction (2018). *Eur Heart J*. 2019;**40**:237-269.

6. Zeitouni M, Silvain J, Guedeney P, Kerneis M, Yan Y, Overtchouk P, Barthelemy O, Hauguel-Moreau M, Choussat R, Helft G, Le FC, Collet JP, Montalescot G. Periprocedural myocardial infarction and injury in elective coronary stenting. *Eur Heart J*. 2018;**39**:1100-1109.

7. Silvain J, Zeitouni M, Paradies V, Zheng HL, Ndrepepa G, Cavallini C, Feldman DN, Sharma SK, Mehilli J, Gili S, Barbato E, Tarantini G, Ooi SY, von BC, Jaffe AS, Thygesen K, Montalescot G, Bulluck H, Hausenloy DJ. Cardiac procedural myocardial injury, infarction, and mortality in patients undergoing elective percutaneous coronary intervention: a pooled analysis of patient-level data. *Eur Heart J*. 2021;**42**:323-334.

8. Koskinas KC, Ndrepepa G, Raber L, Karagiannis A, Kufner S, Zanchin T, Hieber J, Hunziker L, Mayer K, Byrne RA, Heg D, Windecker S, Kastrati A. Prognostic Impact of Periprocedural Myocardial Infarction in Patients Undergoing Elective Percutaneous Coronary Interventions. *Circ Cardiovasc Interv*. 2018;**11**:e006752.

9. Liou K, Jepson N, Kellar P, Ng B, Isbister J, Giles R, Friedman D, Allan R, Lau A, Pitney M, Ooi SY. Prognostic Significance of Peri-procedural Myocardial Infarction in the Era of High Sensitivity Troponin: A Validation of the Joint ACCF/AHA/ESC/WHF Universal Definition of Type 4a Myocardial Infarction with High Sensitivity Troponin T. *Heart Lung Circ*. 2015;**24**:673-681.

10. Feldman DN, Minutello RM, Bergman G, Moussa I, Wong SC. Relation of troponin I levels following nonemergent percutaneous coronary intervention to short- and long-term outcomes. *Am J Cardiol*. 2009;**104**:1210-1215.

11. Chen ZW, Yang HB, Chen YH, Ma JY, Qian JY, Ge JB. Impact of multi-vessel therapy to the risk of periprocedural myocardial injury after elective coronary intervention: exploratory study. *BMC Cardiovasc Disord*. 2017;**17**:69.

12. Di SL, Borgia F, Maeremans J, Pyxaras SA, De BB, Wijns W, Heyndrickx GR, Dens J, Di MC, Barbato E. Periprocedural Myocardial Injury and Long-Term Clinical Outcome in Patients Undergoing Percutaneous Coronary Interventions of Coronary Chronic Total Occlusion. *J Invasive Cardiol*. 2016;**28**:410-414.

13. Loeb HS, Liu JC. Frequency, risk factors, and effect on long-term survival of increased troponin I following uncomplicated elective percutaneous coronary intervention. *Clin Cardiol*. 2010;**33**:E40-E44.

14. Auguadro C, Scalise F, Manfredi M, Casali V, Novelli E, Specchia G. The prognostic role of troponin I elevation after elective percutaneous coronary intervention. *J Cardiovasc Med (Hagerstown )*. 2015;**16**:149-155.

15. Prasad A, Singh M, Lerman A, Lennon RJ, Holmes DR, Jr., Rihal CS. Isolated elevation in troponin T after percutaneous coronary intervention is associated with higher long-term mortality. *J Am Coll Cardiol*. 2006;**48**:1765-1770.

16. Garcia-Garcia HM, McFadden EP, von BC, Rademaker-Havinga T, Spitzer E, Kleiman NS, Cohen DJ, Kennedy KF, Camenzind E, Mauri L, Steg PG, Wijns W, Silber S, van Es GA, Serruys PW, Windecker S, Cutlip D, Vranckx P. Impact of Periprocedural Myocardial Biomarker Elevation on Mortality Following Elective Percutaneous Coronary Intervention. *JACC Cardiovasc Interv*. 2019;**12**:1954-1962.

17. Ndrepepa G, Colleran R, Braun S, Cassese S, Hieber J, Fusaro M, Kufner S, Ott I, Byrne RA, Husser O, Hengstenberg C, Laugwitz KL, Schunkert H, Kastrati A. High-Sensitivity Troponin T and Mortality After Elective Percutaneous Coronary Intervention. *J Am Coll Cardiol*. 2016;**68**:2259-2268.

18. Miller WL, Garratt KN, Burritt MF, Lennon RJ, Reeder GS, Jaffe AS. Baseline troponin level: key to understanding the importance of post-PCI troponin elevations. *Eur Heart J*. 2006;**27**:1061-1069.

19. Prasad A, Rihal CS, Lennon RJ, Singh M, Jaffe AS, Holmes DR, Jr. Significance of periprocedural myonecrosis on outcomes after percutaneous coronary intervention: an analysis of preintervention and postintervention troponin T levels in 5487 patients. *Circ Cardiovasc Interv*. 2008;**1**:10-19.

20. Jeremias A, Kleiman NS, Nassif D, Hsieh WH, Pencina M, Maresh K, Parikh M, Cutlip DE, Waksman R, Goldberg S, Berger PB, Cohen DJ. Prevalence and prognostic significance of preprocedural cardiac troponin elevation among patients with stable coronary artery disease undergoing percutaneous coronary intervention: results from the evaluation of drug eluting stents and ischemic events registry. *Circulation*. 2008;**118**:632-638.

21. Herrmann J, Lennon RJ, Jaffe AS, Holmes DR, Jr., Rihal CS, Prasad A. Defining the optimal cardiac troponin T threshold for predicting death caused by periprocedural myocardial infarction after percutaneous coronary intervention. *Circ Cardiovasc Interv*. 2014;**7**:533-542.

22. Novack V, Pencina M, Cohen DJ, Kleiman NS, Yen CH, Saucedo JF, Berger PB, Cutlip DE. Troponin criteria for myocardial infarction after percutaneous coronary intervention. *Arch Intern Med*. 2012;**172**:502-508.

23. Christensen MK, Huang H, Torp-Pedersen C, Trydal T, Ravkilde J. Incidence and impact on prognosis of peri-procedural myocardial infarction in 2760 elective patients with stable angina pectoris in a historical prospective follow-up study. *BMC Cardiovasc Disord*. 2016;**16**:140.

24. Yang X, Tamez H, Lai C, Ho K, Cutlip D. Type 4a myocardial infarction: Incidence, risk factors, and long-term outcomes. *Catheter Cardiovasc Interv*. 2017;**89**:849-856.

25. Goliasch G, Winter MP, Ayoub M, Bartko PE, Gebhard C, Mashayekhi K, Ferenc M, Buettner HJ, Hengstenberg C, Neumann FJ, Toma A. A Contemporary Definition of Periprocedural Myocardial Injury After Percutaneous Coronary Intervention of Chronic Total Occlusions. *JACC Cardiovasc Interv*. 2019;**12**:1915-1923.

26. De LA, Lemesle G, Bonello L, Syed AI, Collins SD, Ben-Dor I, Pinto Slottow TL, Xue Z, Torguson R, Suddath WO, Satler LF, Kent KM, Pichard AD, Lindsay J, Waksman R. Prognostic significance of small troponin I rise after a successful elective percutaneous coronary intervention of a native artery. *Am J Cardiol*. 2009;**103**:639-645.

27. Cavallini C, Verdecchia P, Savonitto S, Arraiz G, Violini R, Olivari Z, Rubartelli P, De SS, Plebani M, Steffenino G, Sbarzaglia P, Ardissino D. Prognostic value of isolated troponin I elevation after percutaneous coronary intervention. *Circ Cardiovasc Interv*. 2010;**3**:431-435.

28. Jaguszewski M, Gilis-Malinowska N, Gutierrez-Chico JL, Chmielecki M, Skarzynski P, Burakowski S, Drewla P, Targonski R, Lewicki L, Dubaniewicz W, Fijalkowski M, Gruchala M, Ciecwierz D. Periprocedural Myocardial Injury After Recanalization of Single Chronic Coronary Occlusion - A Propensity Score Analysis Comparing Long-Term Clinical Outcomes. *J Invasive Cardiol*. 2017;**29**:63-67.

29. Ferreira RM, de Souza E Silva NA, Salis LHA, da Silva RRM, Maia PD, Horta LFB, Salles EF, Nunes HMP, de Oliveira JBM, Domingues YPS, de Sousa CCM. Troponin I elevation and all-cause mortality after elective percutaneous coronary interventions. *Cardiovasc Revasc Med*. 2017;**18**:255-260.

30. Cottens D, Maeremans J, McCutcheon K, Lamers S, Roux L, Duponselle J, Bennett J, Dens J. Prognostic value of the high-sensitivity troponin T assay after percutaneous intervention of chronic total occlusions. *J Cardiovasc Med (Hagerstown )*. 2018;**19**:366-372.

31. Testa L, van Gaal WJ, Biondi Zoccai GG, Agostoni P, Latini RA, Bedogni F, Porto I, Banning AP. Myocardial infarction after percutaneous coronary intervention: a meta-analysis of troponin elevation applying the new universal definition. *QJM*. 2009;**102**:369-378.

32. Nienhuis MB, Ottervanger JP, Dambrink JH, de Boer MJ, Hoorntje JC, Gosselink AT, Suryapranata H, 't Hof AW. Comparative predictive value of infarct location, peak CK, and ejection fraction after primary PCI for ST elevation myocardial infarction. *Coron Artery Dis*. 2009;**20**:9-14.

33. Feldman DN, Kim L, Rene AG, Minutello RM, Bergman G, Wong SC. Prognostic value of cardiac troponin-I or troponin-T elevation following nonemergent percutaneous coronary intervention: A meta-analysis. *Catheter Cardiovasc Interv*. 2011;**77**:1020-1030.

34. Li Y, Pei H, Bulluck H, Zhou C, Hausenloy DJ. Periprocedural elevated myocardial biomarkers and clinical outcomes following elective percutaneous coronary intervention: a comprehensive dose-response meta-analysis of 44,972 patients from 24 prospective studies. *EuroIntervention*. 2020;**15**:1444-1450.
